# A phase synchronization clustering algorithm for identifying interesting groups of genes from cell cycle expression data

**DOI:** 10.1186/1471-2105-9-56

**Published:** 2008-01-28

**Authors:** Chang Sik Kim, Cheol Soo Bae, Hong Joon Tcha

**Affiliations:** 1 Institute of Animal Resources Research, Kangwon National University, Chuncheon, Republic of Korea; 2 Department of Biomedical Engineering, Kwandong University, Kangnung, Republic of Korea; 3 Department of Information Technology, Kangwon National University, Chuncheon, Republic of Korea

## Abstract

**Background:**

The previous studies of genome-wide expression patterns show that a certain percentage of genes are cell cycle regulated. The expression data has been analyzed in a number of different ways to identify cell cycle dependent genes. In this study, we pose the hypothesis that cell cycle dependent genes are considered as oscillating systems with a rhythm, i.e. systems producing response signals with period and frequency. Therefore, we are motivated to apply the theory of multivariate phase synchronization for clustering cell cycle specific genome-wide expression data.

**Results:**

We propose the strategy to find groups of genes according to the specific biological process by analyzing cell cycle specific gene expression data. To evaluate the propose method, we use the modified *Kuramoto model*, which is a phase governing equation that provides the long-term dynamics of globally coupled oscillators. With this equation, we simulate two groups of expression signals, and the simulated signals from each group shares their own common rhythm. Then, the simulated expression data are mixed with randomly generated expression data to be used as input data set to the algorithm. Using these simulated expression data, it is shown that the algorithm is able to identify expression signals that are involved in the same oscillating process. We also evaluate the method with yeast cell cycle expression data. It is shown that the output clusters by the proposed algorithm include genes, which are closely associated with each other by sharing significant Gene Ontology terms of biological process and/or having relatively many known biological interactions. Therefore, the evaluation analysis indicates that the method is able to identify expression signals according to the specific biological process. Our evaluation analysis also indicates that some portion of output by the proposed algorithm is not obtainable by the traditional clustering algorithm with Euclidean distance or linear correlation.

**Conclusion:**

Based on the evaluation experiments, we draw the conclusion as follows: 1) Based on the theory of multivariate phase synchronization, it is feasible to find groups of genes, which have relevant biological interactions and/or significantly shared GO slim terms of biological process, using cell cycle specific gene expression signals. 2) Among all the output clusters by the proposed algorithm, the cluster with relatively large size has a tendency to include more known interactions than the one with relatively small size. 3) It is feasible to understand the cell cycle specific gene expression patterns as the phenomenon of collective synchronization. 4) The proposed algorithm is able to find prominent groups of genes, which are not obtainable by traditional clustering algorithm.

## Background

Since Hereford *et al*. [1] first discovered yeast histone mRNAs oscillate during cell division cycle, several experimental studies have identified that many genes are expressed in a cell-cycle-specific manner. These studies have motivated the study of global extent of cycle-specific gene expression. To this end, there have been a number of studies using DNA microarrays to understand whole-genome expression patterns during cell division cycle [2-8]. A particular example is flagella biogenesis in *Caulobactor*, which has four distinct and dependent waves of transcription. Laub *et al*. [3] showed that 20% of *Caulobactor *genes are cell cycle regulated, their expression level consistently having peaks when they function. Another example is the study of yeast *Saccharomyces cerevisiae *[6], in which they also discovered that between 10 and 20% of yeast genes are periodically expressed during cell division. Therefore, it suffices to say that a certain percentage of genes may have the periodicity for its oscillatory activity throughout the cell division. These cell-cycle-specific oscillatory activities can be explained by a biological phenomenon in terms of efficiency and logical order. The cell only makes the enzyme when it is needed. If the enzymes were made all the time, the cell would be inefficient in an environment devoid of the substrates of the enzymes [9].

In this study, we are motivated to apply the theory of multivariate phase synchronization to cell-cycle-specific gene expression data. Synchronization is one of the most commonly present phenomena in various fields of science [10,11]. Generally, we understand synchronization as a complete coincidence of the states between oscillating systems due to their interactions. Rosenblum *et al*. [12] show that the phase difference of two coupled oscillating systems is bounded while the amplitude is uncorrelated and irregular. There have been numerous applications in different areas such as cardiorespiratory interaction [13-15], brain activity of Parkinsonian patients [16], EEG measurements [17-20], ecology [21], and climate systems [22]. Because our interests of this study are cellular activity during cell cycle, our interested systems are the cell cycle specific genes. Based on the theory of phase synchronization, we pose a hypothesis that expression signals from two genes could be synchronized if these two genes are biologically interacting with each other. That is, two biologically interacting genes produce oscillating expression signals with a common rhythm. Therefore, we propose the phase synchronization as a measure to identify biologically relevant interactions using cell-cycle-specific gene repression data and the cell cycle specific genes are oscillating systems, which produce gene expressions with rhythms (periodicity).

In this study, we present the effort of applying the theory of multivariate phase synchronization to find groups of cell cyclic gene expression signals according to the specific biological process, which is based on the study of Allefeld and Kurths [17]. They present a method for the multivariate analysis of statistical phase synchronization phenomena in empirical data, which is based on the theory of *synchronization cluster*. The basic idea of their analysis is to consider the oscillating systems forming a cluster in which each one contributes to the cluster in different degree. The cluster consists of a common rhythm that is a mean oscillation for all oscillating systems inside the cluster. Based on their theory, we propose an algorithm named as Phase Synchronization Clustering (PSC) algorithm, which produce the clusters of cell cycle specific genes from genome expression data set, and the genes from the same cluster are expected to be involved in the specific biological process. The PSC algorithm is evaluated with synthetic data and cell cycle specific expression data of *Saccharomyces cerevisiae *from the study of Spellman *et al*. [6], in which they analyze gene expression levels in yeast cell cultures whose cell cycle has been synchronized by various methods.

## Results and discussion

### Case study 1: *in silico *experiments

The purpose of this experiment is to show how the proposed PSC algorithm is able to identify the signals that are expressed in the same specific process. In this study, it is assumed that a certain group of gene expression levels during cell cycle can be explained as the synchronization of large ensembles of oscillators, in which each element of the ensemble interacts with all others and is driven by the mean field that is formed by all elements, provided that every members from the group play a role for a certain biological process. The driving force, or the mean field, is not predetermined, but arises from interactions within the ensemble. This force determines whether the systems synchronize, but it itself depends on their oscillation. We use the modified *Kuramoto model *[23] as a phase governing equation that gives the long-term dynamics of globally coupled oscillators

$$\begin{array}{cc} \frac{d\phi_{i}}{dt}=C\sum_{j=1}^{K}\sin\left(\psi -\phi_{j}\right), & i=1,\ldots ,N, \end{array}$$

where the *φ*_i_s are the instantaneous phase, the *ψ *are the mean phase, and the positive constant C represents the coupling strength. It should be noted that the autonomous (or natural) frequency term is excluded from the original *Kuramoto model *for this model, and the mean phase *ψ *is roughly approximated by averaging the phases of all oscillators at current time point. The original *Kuramoto model *describes a large population of coupled limit-cycle oscillators [23]. With this modified model, it is assumed that the instantaneous rate of phase change is proportional to the mean sinusoidal coupling between the mean phase and each instantaneous phase. Given a set of initial conditions and a step size, we can simulate the instantaneous phase using the following for each gene i

$$\phi_{i}\left(t+1\right)=\phi_{i}\left(t\right)+\delta t\frac{d\phi_{i}}{dt}.$$

With given initial random instantaneous phase signals, the expression signal can be simulated and converted into real signals as

*x*_*i*_(*t*) = *real*[*A *exp[(*jφ*_*i*_(*t*))] = *A *cos(*φ*_*i*_(*t*)),

where A is the instantaneous amplitude and is set to 1 for all signals. Then the simulated signals are updated by adding random noise from Gaussian distribution with mean *μ *= 0 and standard deviation *ε*.

**Figure 1:**
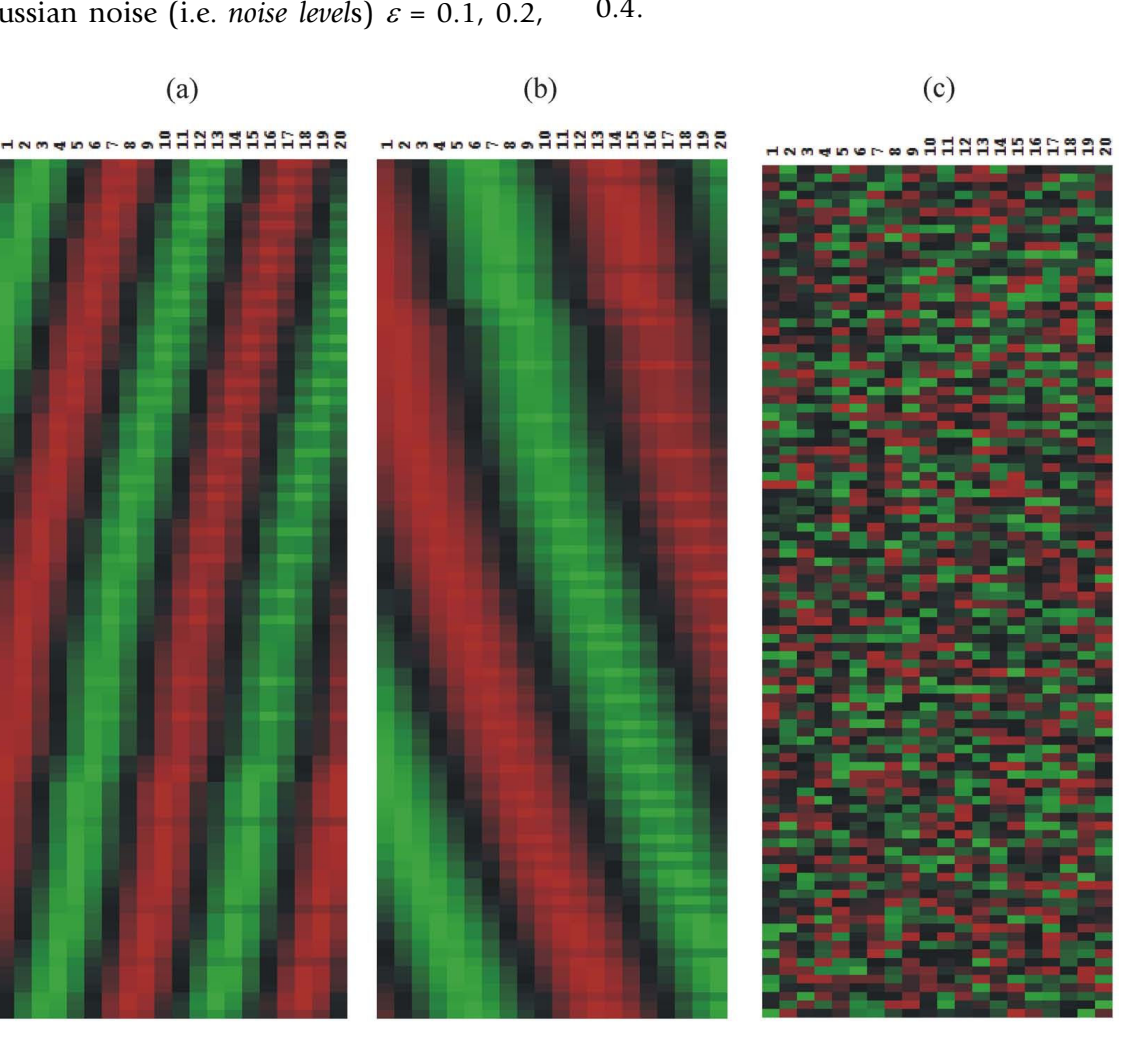
Two groups of simulated expression signals sorted by two most significant principal components p_1 _and p_2 _and a group of random signals: (a) simulated signals with coupling constant C = 3.0 (b) simulated signals with C = 2.0 (c) random signals. The simulated expression signals show traveling waves from 1^st ^time measurement to 20^th ^time measurement. The number of signals for each group is set to 100.

**Figure 2:**
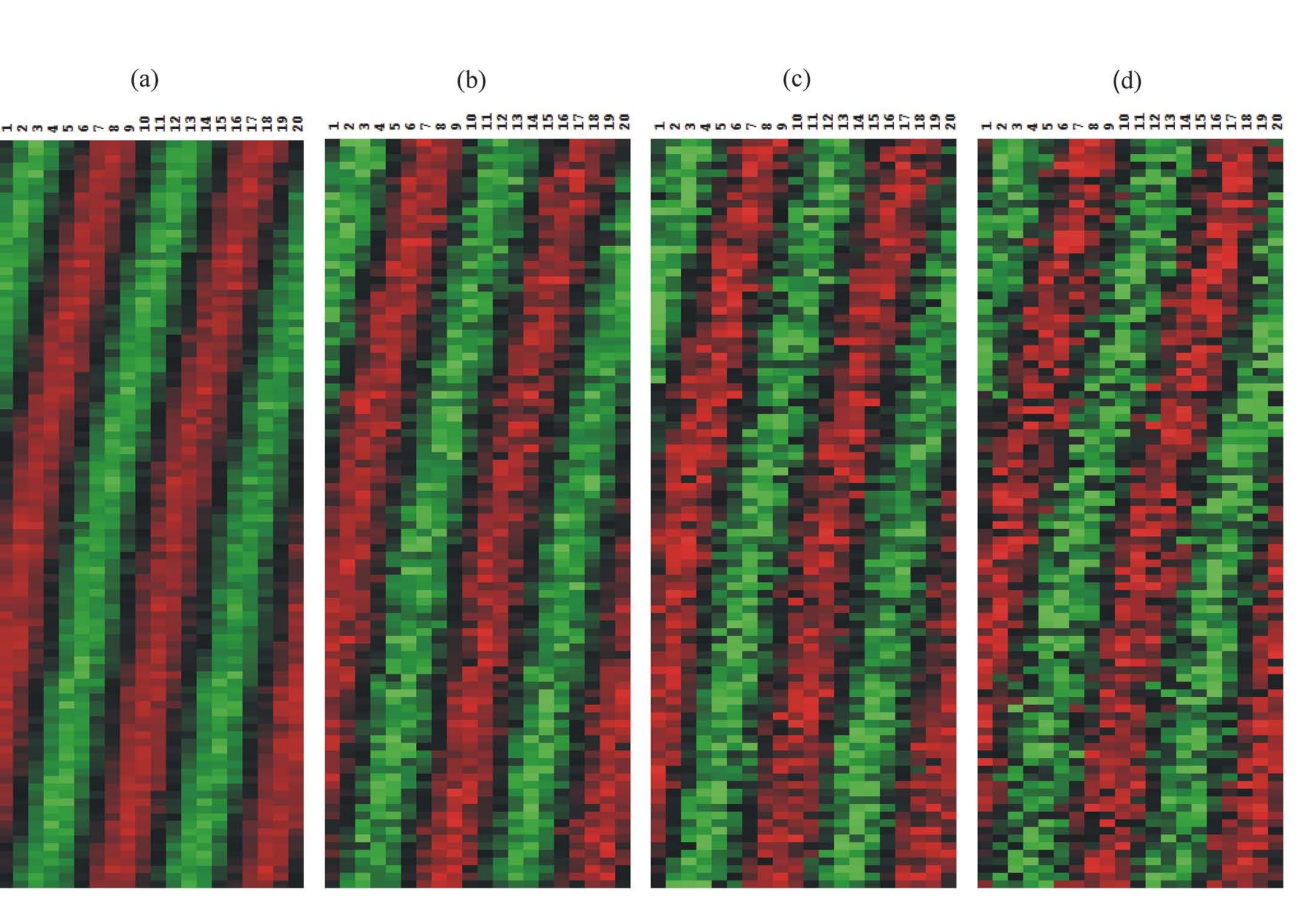
The changes of simulated expression signals with coupling constant C = 3.0 are displayed as the *noise level *increases from 0.1 to 0.4. (a) *noise level ε *= 0.1, (b) *noise level ε *= 0.2, (c) *noise level ε *= 0.3, (d) *noise level ε *= 0.4.

To evaluate the PSC algorithm, we generate four sets of the expression signals with four different standard deviations of random Gaussian noise (i.e. *noise level*s) *ε *= 0.1, 0.2, 0.3, 0.4. For each set, we generate two groups of 100 signals. For first group, 20 measurements of signals are simulated with the coupling strength C = 3.0, and for second group, 20 measurements with the coupling strength C = 2.0. It suffices to say that these two groups are separately involved in their own oscillating process because each group has different coupling strength. It means that each group has different driving forces or mean field for their own signals. For each data set, we generate a group of random signals with same number of genes and measurements. This random group is combined with two other groups of simulated signals. Thus, in each data set, two-thirds of signals are simulated signals and one-third of signals are random signals. Then, we randomly shuffle the locations of all expression signals for each data set. We use four different values of *cutoff *(=0.9, 0.8, 0.7, 0.6) for these four data sets. Figure 1 shows all three groups of sorted expression signals without any addition of noises, and Figure 2 displays the change of the simulated signals of first group (C = 3.0) as the *noise level *increases from 0.1 to 0.4.

As an initial step, the algorithm creates a set of clusters of which the size is equal to the number of signals in the input data set. In this case, the algorithm creates 300 initial clusters, of which all sizes are equal to one. After the final step of the algorithm, the size of each cluster will be different depending on the values of *cutoff *and *noise level ε*. For each non-empty cluster, the signals from the group with simulated signals are counted and labeled as true positive (TP) for each group, and the signals from the group with random signals are also counted and labeled as false positive (FP).

**Figure 3:**
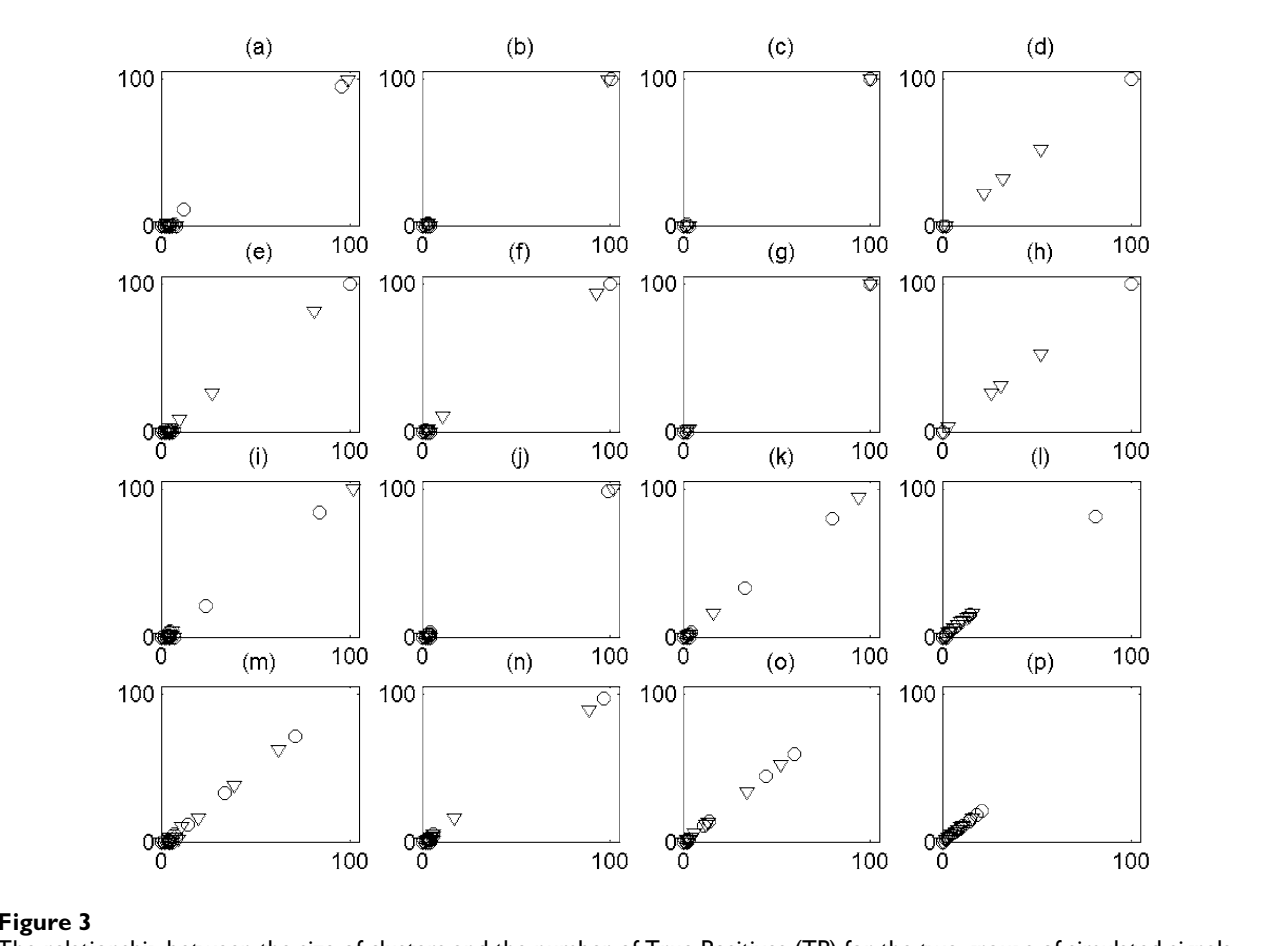
The relationship between the size of clusters and the number of True Positives (TP) for the two groups of simulated signals with four different values of *cutoff *and *noise level ε*. For all figures, the x-axis corresponds to the size of clusters, and the y-axis the number of TP. The *circle *corresponds to the cluster that consists of signals from the first group, and the *triangle *the ones from the second group. The expression signals of first group are simulated with coupling constant C = 3.0, and the ones of second group with C = 2.0. See Table 7 for the index of each figure depending on *cutoff *and *noise level ε*.

**Figure 4:**
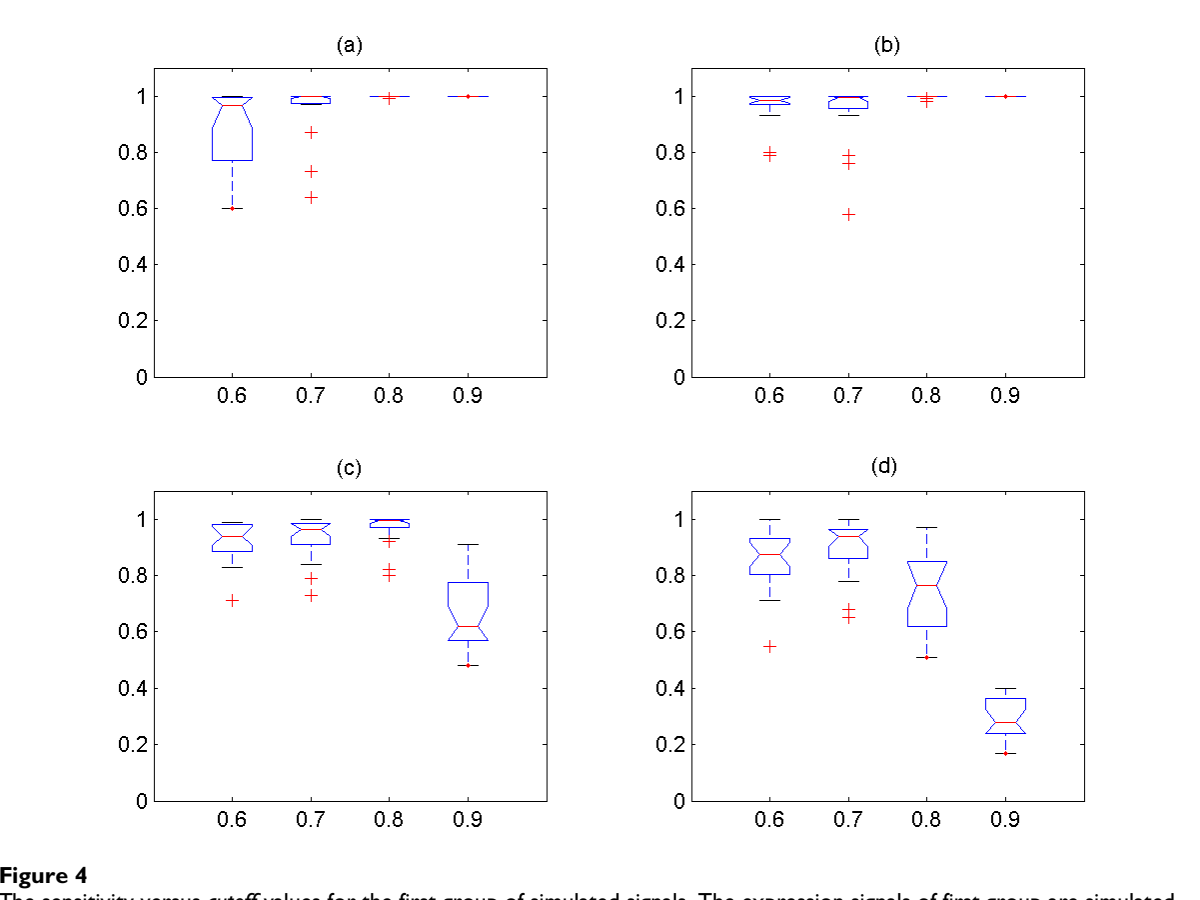
The sensitivity versus *cutoff *values for the first group of simulated signals. The expression signals of first group are simulated with coupling constant C = 3.0. (a) *noise level ε *= 0.1, (b) *noise level ε *= 0.2, (c) *noise level ε *= 0.3, (d) *noise level ε *= 0.4. For all figures, the x-axis corresponds to the *cutoff *values, and the y-axis the sensitivity.

**Figure 5:**
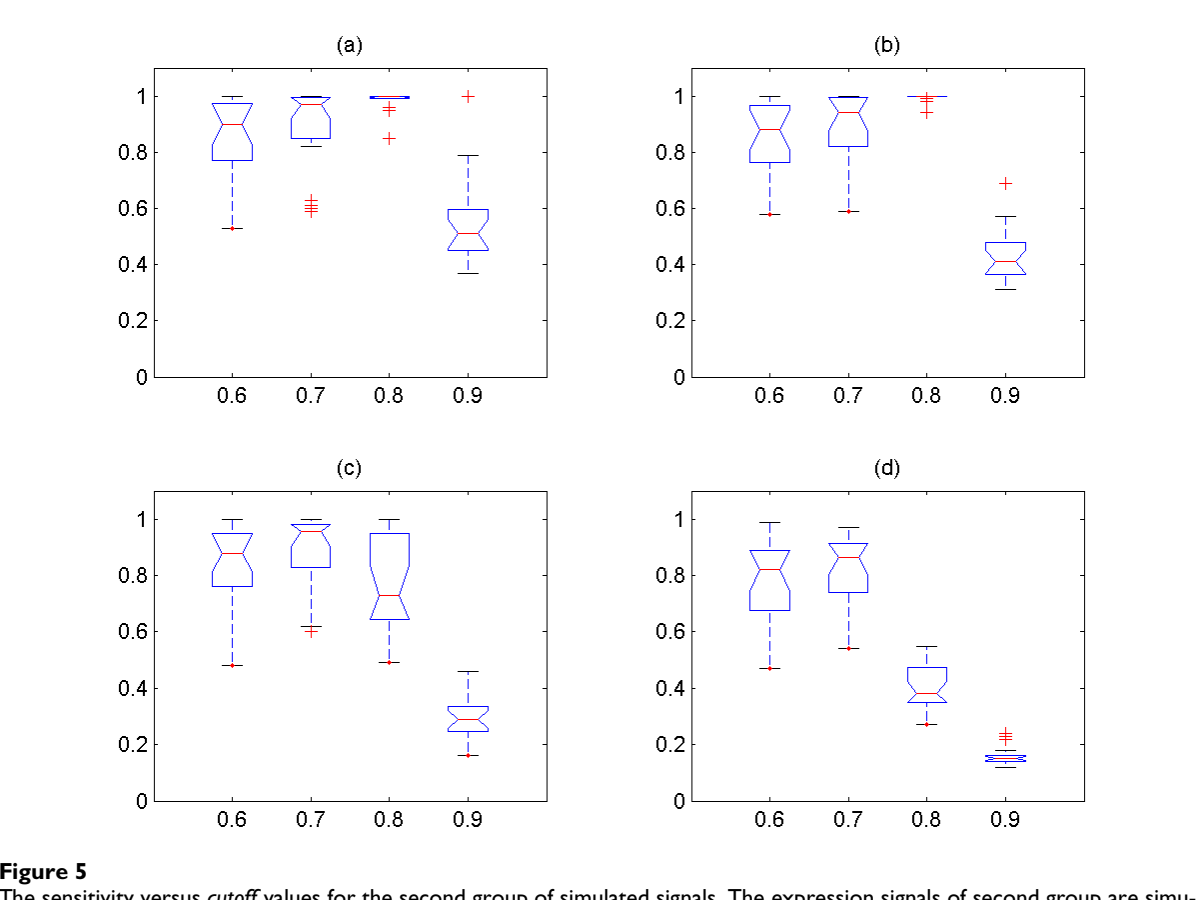
The sensitivity versus *cutoff *values for the second group of simulated signals. The expression signals of second group are simulated with coupling constant C = 2.0. (a) *noise level ε *= 0.1, (b) *noise level ε *= 0.2, (c) *noise level ε *= 0.3, (d) *noise level ε *= 0.4. For all figures, the x-axis corresponds to the *cutoff *values, and the y-axis the sensitivity.

**Figure 6:**
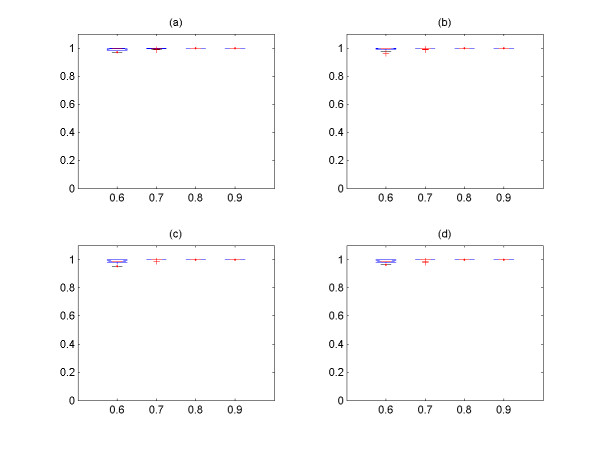
The precision versus *cutoff *values for the first group of simulated signals. The expression signals of first group are simulated with coupling constant C = 3.0. (a) *noise level ε *= 0.1, (b) *noise level ε *= 0.2, (c) *noise level ε *= 0.3, (d) *noise level ε *= 0.4. For all figures, the x-axis corresponds to the *cutoff *values, and the y-axis the precision.

**Figure 7:**
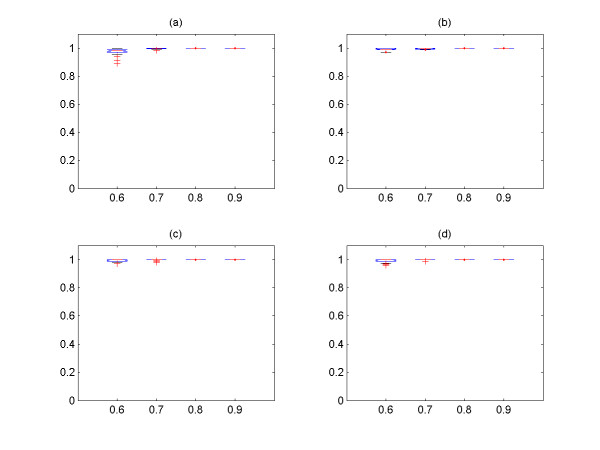
The precision versus *cutoff *values for the second group of simulated signals. The expression signals of second group are simulated with coupling constant C = 2.0. (a) *noise level ε *= 0.1, (b) *noise level ε *= 0.2, (c) *noise level ε *= 0.3, (d) *noise level ε *= 0.4. For all figures, the x-axis corresponds to the *cutoff *and the y-axis the precision.

In Figure 3, we explore the relationship between the size of clusters and the number of TP from the two groups of simulated signals with four different values of *cutoff *and *noise level ε*, and it is shown that all of them have linear relationship. It is also shown that the 1^st ^and 2^nd ^largest clusters contain the most TP signals among the output clusters. Hence, only these two largest output clusters are used as the output of the algorithm, and we explore the effect of the *cutoff *and *noise level *on the performance of the algorithm with these two clusters. We then systemically compare the sensitivity (percentage of correctly identified from input expression signals) and precision (percentage of TP expression signals among the output expression signals) for different *cutoff *and *noise level*s *ε *(Figure 4, 5, 6, 7) See Table 7 for the index of each figure. To test the variability of the results, we run the algorithm 20 times for each value of *cutoff *and *noise level ε*. Note that the simulated expression signals are different for each run due to random generations of initial phase signals and random noise addition, and the results are also expected to have certain degree of variability.

It is shown that the more noises are included in the data set, the less the sensitivity is obtained by the method (Figure 4, 5). On the other hand, the overall precision is almost constant (i.e. = 100%) as the *noise level ε *increases (Figure 6, 7), i.e. the almost 100% of the output signals are TP signals. It is shown that the sensitivity are approximately 82 – 96% with *cutoff *= 0.7 for all *noise level*s. If we assume that the *noise level ε *is ≤0.4, the *cutoff *values to obtain the sensitivity ≥82% for both groups should be 0.7. Based on this experiment, we conclude that the *cutoff *value ≥ 0.7 should be used for the analysis of yeast expression data to evaluate the PSC method, provided that the *noise level *in yeast data is ≤0.4. This could be reasonable assumption, because it is believed that the *noise level *= 0.4 is relatively large.

### Case study 2: *α *factor-synchronized cell cycle gene expression data analysis

We evaluate the PSC algorithm with expression data from the study of Spellman *et al*. [6], in which they monitor genome-wide mRNA levels by using four different cell cycle synchronization techniques. We evaluate the PSC algorithm with the data sets by three yeast experiments (Alpha, Cdc15, and Cdc28), in which mRNA levels of 6,178 yeast ORFs are measured simultaneously over approximately two cell cycle periods. A fourth data set using elutriation-based experiment by Spellman *et al*. [6] is not used, since it only covers a single cell cycle and because most published methods were not applied to this data set. There are many missing values in Spellman *et al*. [6]'s data set – only 605 genes have no missing values in all three data sets. These missing values can lead to problems in the data analysis, because they obviously interfere with computation of any statistical test or clustering. The one of default ways to handle these missing values is to exclude all data points that have missing data in at least one of the selected genes. However, if missing data points are randomly distributed across the arrays, there could be very few "valid" data points left to be analyzed in the data sets, because of the abundance of missing values in our chosen data sets. Therefore, we replace the missing values for each gene by the mean expression levels of its gene. We perform mean imputation in the gene expression levels for the *K *= 4,201 genes, which have no more than one missing value in each expression data set. Then the expression profile is normalized to the standardized variable. Let's say we have an expression profile *z(t)*, *t *= 1, 2, ..., *n*. If the expression profile has mean *μ *and variance *σ*^2^, then the corresponding normalized expression value

$$x(t)=\frac{z(t)-\mu }{\sigma }$$

has the mean 0 and variance 1.

It is clear that each expression data set contains artifacts, which would not occur in freely growing cells, due to the treatment of the cells for the cell cycle synchronization. For example, in Cdc15 and Cdc28 experiments, cells are released from arrest by an abrupt drop in temperature, which likely results in changes in expression of e.g. heat shock genes. To avoid the artifacts due to each cell cycle synchronization treatment, three expression data sets should be combined to uncover the "correct" sets of genes using the PSC algorithm as follows. The PSC algorithm mainly consists of the estimation of the strength between a system and the cluster, *ρ*_*aC *_in Eq. (9). For the estimation of *ρ*_*aC*_, the algorithm requires the strength values of the bivariate synchronization between all systems within the cluster of interest to us, *ρ*_*a*,*b *_in Eq. (4). As a step for combining three data sets, we calculate the average *ρ*_*a*,*b *_values from the three data sets as follows

$$\rho_{a,b}=\frac{1}{3}\left[\rho_{a,b}^{\alpha }+\rho_{a,b}^{cdc15}+\rho_{a,b}^{cdc28}\right],$$

where *ρ*_*a*,*b*_^*α *^is the values of bivariate synchronization by alpha-factor data set, *ρ*_*a*,*b*_^*cdc*15 ^by cdc15 data set, and *ρ*_*a*,*b*_^*cdc*28 ^by cdc28 data set. It is noteworthy that this step could also reduce the noises in expression data due to the missing values.

The main purpose of the PSC algorithm is to find groups of genes according to the specific biological process using the cell-cycle-specific expression data. The previous *in silico *experiment provide the effectiveness of the PSC algorithm to reach this purpose. Based on the theory of multivariate phase synchronization [17], it is assumed that each gene from the same output cluster is closely associated by having relevant biological interactions and/or sharing significant Gene Ontology (GO) terms. That is, each cluster is related with a certain specific biological process. To evaluate the PSC algorithm, all of output clusters with different *cutoff *= 0.9, 0.8, 0.7, 0.6, 0.5 are analyzed with the *GO Term Finder*. It is a tool for searching significant GO terms, or parent GO terms, used to annotate genes in a given cluster and is available from *Saccharomyces *Genome Database [24]. The significant GO terms for each output clusters depending on *cutoff *are presented as Additional files 1, 2, 3, 4, 5 and the summary of the GO analysis is also provided in Table 1. Note that the *p-value *cutoff for significant GO terms is set to 0.05. It is found that significant number of clusters from the output have significant GO terms. It should be noted that genes within those significant clusters are evidently associated with certain specific biological processes depending on the GO terms of their own.

**Table 1:** The result from the analysis for significant GO terms according to the *cutoff *value.

*cutoff*	*n*1	*n*2	Tables of significant GO terms for the output clusters
0.9	8	10	See Additional file 1
0.8	57	109	See Additional file 2
0.7	148	417	See Additional file 3
0.6	151	447	See Additional file 4
0.5	21	57	See Additional file 5

*n*1 corresponds to the number of output clusters that have at least one significant GO terms and *n*2 the total number of output clusters.

As another evaluation experiment, experimentally identified physical and genetic interactions between genes are mined from *BioGRID *database for each cluster from the output. Note that the *BioGRID *database is a freely accessible database of physical and genetic interactions available at [25]. The numbers of known interactions are presented for clusters only with significant GO terms in Table 2, 3, 4, 5, 6. Note that the clusters are sorted according the size of clusters for each output. It is noticed that the relatively large clusters tend to have many known biological interactions. It means that the genes within relatively large clusters are evidently interacting each other during cell division cycle by participating in the certain specific biological process depending on the significant GO terms of their own. Therefore, it suffices to say that the relatively large clusters are prominent clusters among the output clusters.

**Table 2:** The number of known biological interactions mined from *BioGRID *database [25] for each output cluster with *cutoff *= 0.9.

***ci***	***n1***	***n2***	***n3***	***size***	*ci*	*n*1	*n*2	*n*3	*size*
**1**	**4**	**4**	**12**	**12**	5	0	0	5	5
**2**	**3**	**4**	**7**	**7**	6	0	0	4	4
**3**	**0**	**0**	**6**	**6**	9	0	0	4	4
**4**	**5**	**4**	**5**	**5**	10	6	4	4	4

*ci *corresponds to the cluster index, *n*1 the number of known biological interactions, *n*2 the number of genes within each cluster having interactions with other genes, *n*3 the number of known cell-cycle regulated genes according to the Spellman *et al*. [6], and *size *the number of genes within each cluster.

**Table 3:** The number of known biological interactions mined from *BioGRID *database [25] for each output cluster with *cutoff *= 0.8.

***ci***	***n1***	***n2***	***n3***	***size***	*ci*	*n*1	*n*2	*n*3	*size*	***ci***	***n1***	***n2***	***n3***	***size***	*ci*	*n*1	*n*2	*n*3	*size*
**2**	**2**	**2**	**16**	**16**	19	1	1	2	7	**47**	**3**	**3**	**0**	**5**	80	0	0	0	4
**3**	**5**	**5**	**14**	**14**	22	0	0	7	7	**48**	**1**	**1**	**0**	**5**	83	2	3	3	4
**4**	**2**	**2**	**11**	**11**	23	0	0	7	7	**49**	**3**	**3**	**0**	**5**	86	1	1	0	4
**5**	**1**	**1**	**11**	**11**	26	4	4	0	6	**61**	**2**	**2**	**0**	**4**	91	0	0	0	4
**6**	**0**	**0**	**11**	**11**	27	2	2	0	6	**63**	**0**	**0**	**2**	**4**	92	1	1	0	4
**7**	**0**	**0**	**9**	**9**	28	4	4	6	6	**66**	**1**	**2**	**4**	**4**	95	2	2	4	4
**9**	**6**	**5**	**9**	**9**	32	0	0	5	6	**67**	**1**	**1**	**4**	**4**	96	0	0	4	4
**10**	**2**	**3**	**8**	**8**	33	2	2	6	6	**68**	**1**	**2**	**3**	**4**	100	2	2	1	4
**11**	**8**	**6**	**8**	**8**	37	7	5	0	5	**69**	**2**	**2**	**0**	**4**	103	0	0	0	4
**12**	**1**	**1**	**8**	**8**	38	0	0	5	5	**70**	**1**	**1**	**0**	**4**	104	0	0	0	4
**13**	**1**	**2**	**8**	**8**	39	0	0	5	5	**73**	**1**	**1**	**0**	**4**	106	0	0	2	4
**14**	**1**	**1**	**8**	**8**	41	1	1	1	5	**74**	**1**	**1**	**0**	**4**	109	0	0	1	4
**15**	**1**	**1**	**7**	**7**	44	3	3	0	5	**75**	**0**	**0**	**4**	**4**					
**16**	**2**	**3**	**7**	**7**	45	3	3	0	5	**76**	**1**	**2**	**0**	**4**					
**18**	**2**	**2**	**6**	**7**	46	0	0	0	5	**79**	**2**	**2**	**0**	**4**					

*ci *corresponds to the cluster index, *n*1 the number of known biological interactions, *n*2 the number of genes within each cluster having interactions with other genes, *n*3 the number of known cell-cycle regulated genes according to the classification by Spellman *et al*. [6], and *size *the number of genes within each cluster.

**Table 4:** The number of known biological interactions mined from *BioGRID *database [25] for each output cluster with *cutoff *= 0.7.

**ci**	**n1**	**n2**	**n3**	**size**	ci	n1	n2	n3	size	**ci**	**n1**	**n2**	**n3**	**size**	ci	n1	n2	n3	size
**1**	**24**	**19**	**50**	**50**	51	0	0	2	10	**138**	**2**	**3**	**0**	**7**	283	0	0	0	6
**2**	**25**	**25**	**44**	**44**	53	3	4	5	10	**140**	**2**	**2**	**0**	**7**	284	3	3	0	6
**3**	**14**	**12**	**8**	**31**	56	1	1	0	10	**149**	**0**	**0**	**0**	**7**	285	1	2	0	6
**4**	**14**	**15**	**25**	**27**	57	3	3	4	10	**150**	**2**	**2**	**3**	**7**	292	2	2	1	6
**5**	**3**	**5**	**21**	**24**	58	0	0	3	10	**151**	**2**	**2**	**0**	**7**	297	0	0	0	6
**6**	**3**	**3**	**10**	**24**	60	4	3	7	10	**154**	**1**	**1**	**0**	**7**	305	3	3	0	6
**9**	**14**	**9**	**1**	**18**	61	0	0	5	10	**155**	**2**	**2**	**2**	**7**	306	2	2	1	6
**10**	**4**	**4**	**17**	**18**	62	1	1	0	9	**160**	**0**	**0**	**1**	**7**	307	3	2	4	6
**11**	**7**	**4**	**18**	**18**	66	3	3	8	9	**164**	**2**	**2**	**0**	**7**	309	8	6	0	6
**12**	**8**	**6**	**11**	**17**	69	1	1	3	9	**168**	**0**	**0**	**2**	**7**	313	1	1	2	6
**13**	**4**	**5**	**15**	**17**	72	2	3	2	9	**173**	**8**	**5**	**5**	**7**	315	1	1	0	6
**14**	**2**	**3**	**17**	**17**	76	5	6	0	9	**175**	**2**	**2**	**4**	**7**	320	1	1	0	6
**16**	**7**	**5**	**3**	**16**	77	0	0	1	9	**177**	**2**	**3**	**0**	**7**	321	0	0	0	6
**17**	**10**	**11**	**13**	**16**	78	2	3	6	9	**181**	**1**	**1**	**0**	**7**	322	3	3	0	6
**18**	**19**	**12**	**0**	**16**	82	1	1	8	9	**182**	**1**	**1**	**1**	**7**	334	1	1	1	5
**19**	**1**	**1**	**2**	**16**	83	3	5	1	9	**185**	**2**	**2**	**0**	**7**	347	3	3	0	5
**20**	**4**	**6**	**0**	**15**	87	0	0	0	8	**190**	**0**	**0**	**4**	**6**	348	1	1	2	5
**21**	**3**	**3**	**8**	**14**	91	4	4	0	8	**204**	**1**	**2**	**0**	**6**	349	2	2	0	5
**22**	**2**	**2**	**14**	**14**	93	0	0	5	8	**205**	**1**	**2**	**0**	**6**	353	0	0	0	5
**23**	**8**	**7**	**0**	**14**	94	0	0	2	8	**206**	**2**	**2**	**0**	**6**	356	2	3	0	5
**24**	**0**	**0**	**13**	**13**	96	1	1	2	8	**209**	**4**	**4**	**0**	**6**	359	0	0	1	5
**26**	**5**	**6**	**0**	**13**	99	3	3	0	8	**210**	**0**	**0**	**0**	**6**	360	1	1	0	5
**28**	**2**	**3**	**0**	**12**	100	0	0	0	8	**211**	**1**	**1**	**2**	**6**	368	2	2	0	5
**29**	**0**	**0**	**3**	**12**	101	11	6	0	8	**218**	**0**	**0**	**0**	**6**	374	1	1	2	5
**31**	**1**	**1**	**0**	**12**	102	5	4	0	8	**228**	**5**	**4**	**2**	**6**	387	2	2	1	5
**34**	**1**	**2**	**5**	**12**	103	0	0	5	8	**229**	**1**	**2**	**4**	**6**	388	2	2	2	5
**35**	**4**	**4**	**0**	**11**	104	3	3	0	8	**230**	**0**	**0**	**0**	**6**	389	0	0	0	5
**36**	**3**	**3**	**0**	**11**	105	4	4	1	8	**233**	**1**	**1**	**0**	**6**	393	2	3	4	5
**39**	**2**	**2**	**1**	**11**	106	0	0	0	8	**235**	**1**	**1**	**0**	**6**	395	0	0	5	5
**41**	**23**	**9**	**0**	**11**	107	0	0	3	8	**239**	**2**	**2**	**0**	**6**	396	3	3	0	5
**42**	**4**	**6**	**1**	**11**	113	0	0	5	8	**243**	**0**	**0**	**0**	**6**	397	1	1	0	5
**43**	**2**	**2**	**0**	**11**	119	0	0	8	8	**244**	**1**	**2**	**0**	**6**	401	0	0	0	5
**44**	**4**	**4**	**0**	**11**	120	3	3	4	8	**246**	**2**	**2**	**0**	**6**	403	1	1	0	5
**45**	**1**	**1**	**1**	**11**	125	1	1	2	8	**263**	**1**	**1**	**0**	**6**	413	1	2	0	5
**46**	**4**	**5**	**8**	**10**	127	0	0	0	8	**274**	**1**	**1**	**1**	**6**	414	1	2	1	5
**48**	**1**	**1**	**2**	**10**	128	2	3	0	8	**275**	**2**	**2**	**0**	**6**	415	1	1	0	5
**50**	**2**	**2**	**1**	**10**	130	4	4	0	8	**278**	**1**	**1**	**0**	**6**	417	1	2	0	5

*ci *corresponds to the cluster index, *n*1 the number of known biological interactions, *n*2 the number of genes within each cluster having interactions with other genes, *n*3 the number of known cell-cycle regulated genes according to the Spellman *et al*. [6], and *size *the number of genes within each cluster.

**Table 5:** The number of known biological interactions mined from *BioGRID *database [25] for each output cluster with *cutoff *= 0.6.

**ci**	**n1**	**n2**	**n3**	**size**	ci	n1	n2	n3	size	**ci**	**n1**	**n2**	**n3**	**size**	ci	n1	n2	n3	size
**1**	**109**	**58**	**108**	**118**	80	2	3	0	20	**184**	**5**	**5**	**4**	**13**	297	1	2	2	9
**2**	**108**	**61**	**105**	**116**	84	3	3	0	19	**186**	**2**	**2**	**1**	**13**	307	0	0	1	8
**3**	**32**	**31**	**19**	**88**	86	1	1	7	19	**187**	**1**	**1**	**0**	**13**	312	0	0	0	8
**4**	**24**	**23**	**32**	**83**	91	3	4	0	19	**188**	**2**	**2**	**0**	**13**	313	0	0	0	8
**5**	**104**	**41**	**1**	**69**	97	6	6	0	18	**196**	**2**	**2**	**2**	**13**	315	1	2	1	8
**9**	**19**	**20**	**33**	**43**	100	8	10	0	18	**197**	**0**	**0**	**1**	**13**	316	2	2	1	8
**11**	**13**	**12**	**8**	**43**	108	7	7	0	18	**198**	**0**	**0**	**1**	**13**	325	2	2	0	8
**12**	**11**	**10**	**34**	**41**	112	7	6	0	18	**199**	**2**	**2**	**0**	**13**	330	0	0	0	8
**15**	**16**	**14**	**20**	**35**	113	0	0	17	18	**202**	**1**	**1**	**5**	**12**	332	1	1	0	8
**17**	**15**	**13**	**0**	**33**	116	4	5	0	17	**206**	**2**	**2**	**1**	**12**	334	5	4	0	8
**19**	**96**	**22**	**0**	**32**	117	3	5	2	17	**207**	**5**	**6**	**1**	**12**	340	1	1	1	8
**20**	**15**	**13**	**2**	**30**	120	5	4	2	17	**213**	**1**	**1**	**5**	**12**	354	2	3	0	8
**22**	**4**	**4**	**0**	**30**	121	5	5	0	17	**214**	**3**	**3**	**1**	**12**	355	5	5	0	8
**27**	**7**	**8**	**2**	**29**	122	2	3	14	16	**215**	**1**	**1**	**4**	**12**	356	2	2	0	8
**30**	**18**	**12**	**0**	**28**	124	1	1	7	16	**217**	**2**	**2**	**0**	**12**	358	0	0	3	8
**31**	**8**	**12**	**6**	**28**	127	0	0	2	16	**219**	**0**	**0**	**0**	**12**	364	1	1	0	8
**34**	**3**	**3**	**11**	**27**	129	0	0	0	16	**221**	**2**	**3**	**0**	**12**	365	2	2	0	8
**36**	**7**	**7**	**9**	**26**	132	4	4	7	16	**222**	**1**	**1**	**3**	**12**	366	1	1	1	8
**37**	**25**	**16**	**0**	**26**	134	4	5	3	16	**230**	**5**	**6**	**0**	**11**	372	2	3	0	7
**38**	**4**	**5**	**1**	**26**	137	4	6	0	16	**231**	**1**	**1**	**0**	**11**	374	3	3	1	7
**39**	**7**	**8**	**0**	**26**	138	5	7	1	16	**232**	**1**	**1**	**2**	**11**	375	0	0	0	7
**40**	**4**	**4**	**1**	**26**	139	3	4	3	16	**236**	**0**	**0**	**2**	**11**	377	2	2	0	7
**41**	**4**	**4**	**2**	**26**	140	3	3	0	15	**237**	**2**	**2**	**0**	**11**	378	2	2	0	7
**42**	**5**	**5**	**13**	**26**	143	1	2	0	15	**240**	**0**	**0**	**2**	**11**	388	0	0	2	6
**44**	**17**	**12**	**18**	**26**	145	4	4	3	15	**241**	**7**	**5**	**0**	**11**	389	0	0	1	6
**46**	**2**	**2**	**10**	**25**	147	9	9	0	15	**242**	**2**	**2**	**2**	**11**	395	1	1	2	6
**50**	**31**	**11**	**2**	**24**	150	2	2	0	15	**243**	**3**	**3**	**0**	**11**	413	1	1	0	6
**51**	**3**	**3**	**22**	**23**	151	6	6	2	15	**245**	**4**	**4**	**0**	**11**	416	0	0	0	6
**52**	**23**	**13**	**10**	**23**	152	0	0	1	15	**249**	**2**	**2**	**0**	**10**	420	0	0	0	6
**53**	**4**	**4**	**0**	**23**	153	2	2	0	14	**255**	**3**	**3**	**0**	**10**	421	0	0	0	6
**54**	**8**	**10**	**2**	**23**	154	4	5	0	14	**259**	**2**	**3**	**0**	**10**	429	1	1	0	6
**58**	**4**	**3**	**0**	**22**	155	1	2	3	14	**267**	**4**	**5**	**2**	**10**	431	1	1	0	6
**63**	**7**	**8**	**0**	**22**	166	2	2	0	14	**270**	**1**	**1**	**2**	**10**	433	1	1	0	6
**67**	**1**	**1**	**3**	**21**	167	2	2	2	14	**285**	**3**	**3**	**0**	**10**	441	2	2	0	6
**71**	**7**	**7**	**12**	**21**	169	10	6	0	14	**289**	**1**	**1**	**2**	**10**	442	3	3	0	6
**74**	**12**	**12**	**1**	**20**	175	4	5	1	14	**290**	**0**	**0**	**0**	**10**	444	1	1	0	6
**76**	**2**	**2**	**9**	**20**	176	1	1	0	14	**291**	**6**	**4**	**0**	**10**	445	0	0	0	6
**79**	**3**	**3**	**0**	**20**	180	4	4	0	13	**296**	**4**	**4**	**0**	**9**					

*ci *corresponds to the cluster index, *n*1 the number of known biological interactions, *n*2 the number of genes within each cluster having interactions with other genes, *n*3 the number of known cell-cycle regulated genes according to the Spellman *et al*. [6], and *size *the number of genes within each cluster.

**Table 6:** The number of known biological interactions mined from *BioGRID *database [25] for each output cluster with *cutoff *= 0.5.

***ci***	***n1***	***n2***	***n3***	***size***	*ci*	*n*1	*n*2	*n*3	*size*	***ci***	***n1***	***n2***	***n3***	***size***	*ci*	*n*1	*n*2	*n*3	*size*
**1**	**483**	**244**	**30**	**429**	11	133	88	1	170	**27**	**48**	**39**	**4**	**98**	46	11	14	6	57
**2**	**464**	**165**	**242**	**277**	13	73	61	14	159	**29**	**56**	**44**	**5**	**92**	50	16	13	2	51
**4**	**187**	**113**	**30**	**245**	14	257	78	13	150	**32**	**33**	**31**	**7**	**81**	54	10	11	1	33
**6**	**146**	**110**	**15**	**212**	15	69	57	36	148	**39**	**22**	**21**	**2**	**68**					
**7**	**185**	**107**	**12**	**204**	16	72	55	86	147	**43**	**8**	**11**	**4**	**63**					
**8**	**63**	**54**	**19**	**184**	24	80	59	1	116	**45**	**8**	**11**	**5**	**58**					

*ci *corresponds to the cluster index, *n*1 the number of known biological interactions, *n*2 the number of genes within each cluster having interactions with other genes, *n*3 the number of known cell-cycle regulated genes according to the Spellman *et al*. [6], and *size *the number of genes within each cluster.

The traditional clustering algorithms focus on relationships based on similar expression profiles, identifying cluster of genes whose expression signals simultaneously rise or fall with an assumption that genes with similar expression profiles have similar biological functions. For example, Spellman *et al*. [6] identify a large number of genes (~800) as giving a cell-cycle-specific patterns of gene expression by fitting the expression profile of given gene to a sine wave, which is used as a surrogate pattern of ideal cyclicity. Then, they use the hierarchical clustering algorithm to linearly correlate the expression profile for a given gene with the expression profile of other genes, which are considered to be confirmed as certain cell-cycle-regulated genes. To this end, they cluster genes into five cell cycle phases (G1, S, S/G2, G2/M, and M/G1). On the other hand, the PSC algorithm use the theory of multivariate phase synchronization, in which the mean phase coherence in Eq. 4 are used to find closely related genes that have relevant biological interactions and/or sharing significant GO terms. Here, the PSC algorithm deal with a special case of random variable that is defined on a circular scale, such that values whose difference is an integral multiple of a certain period (i.e. 2*π*) are regarded the same, and all values are wrapped into a single period. Note that the phase difference between expression profiles (or the phase of a expression profile) is an example of circular random variables *φ*_*i *_(i = 1, 2,...). It is noteworthy that standard (or linear) statistical measures and moments like mean and variance are not applicable, because they yield different values if the period is added to or subtracted from some values, though the physical meaning of these changed values is the same. Based on the theory of phase synchronization, it is assumed that expression signals from two genes could be synchronized if these two genes are biologically interacting with each other. That is, two biologically interacting genes produce oscillating expression signals with a common rhythm. This phenomenon is explained in terms of coincidence of frequencies defined as "phase locking" [12]. With this theory, it is possible to measure the coupling strength between genes, which describes how strong the interaction is between genes.

**Figure 8:**
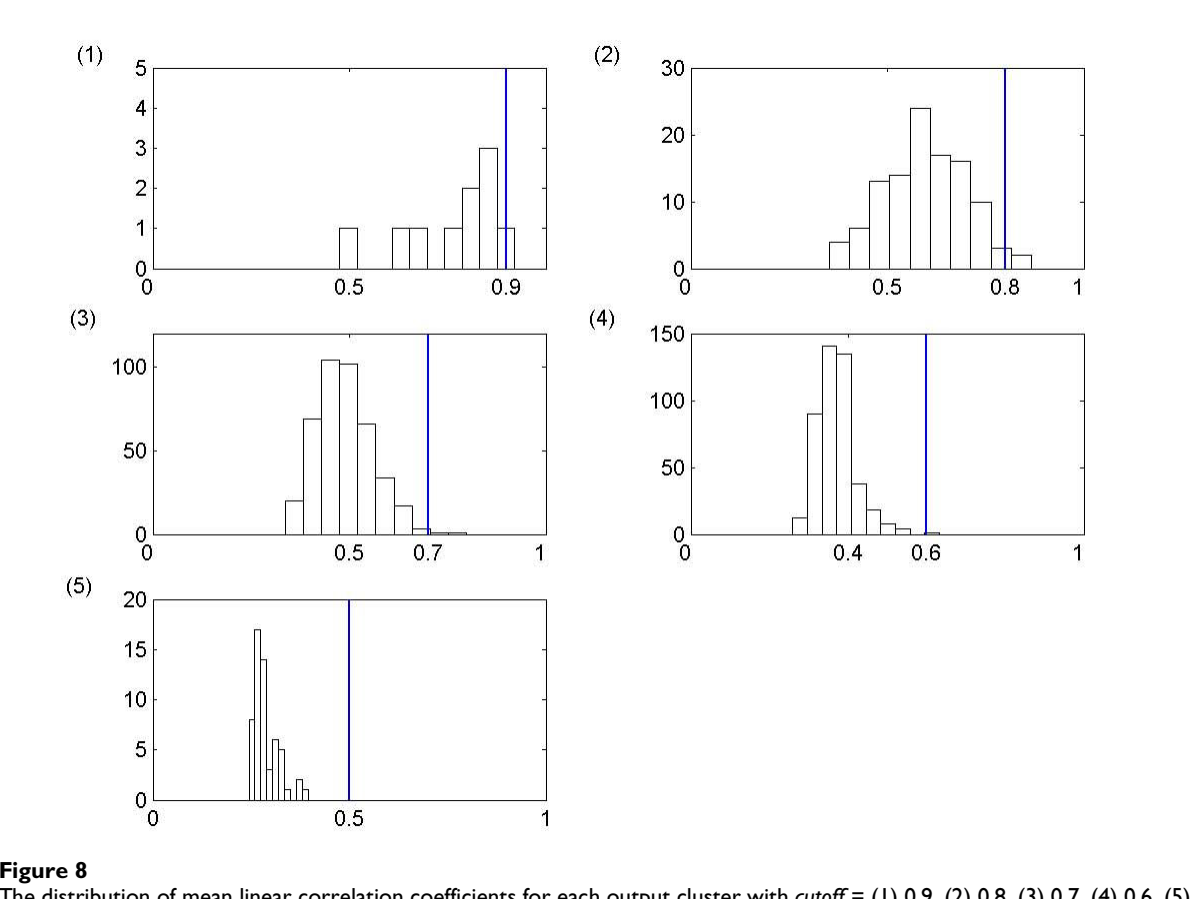
The distribution of mean linear correlation coefficients for each output cluster with *cutoff *= (1) 0.9, (2) 0.8, (3) 0.7, (4) 0.6, (5) 0.5. For all figures, the x-axis corresponds to linear correlation coefficient and the y-axis the number of output clusters that fall within the range of linear correlation coefficient.

**Figure 9:**
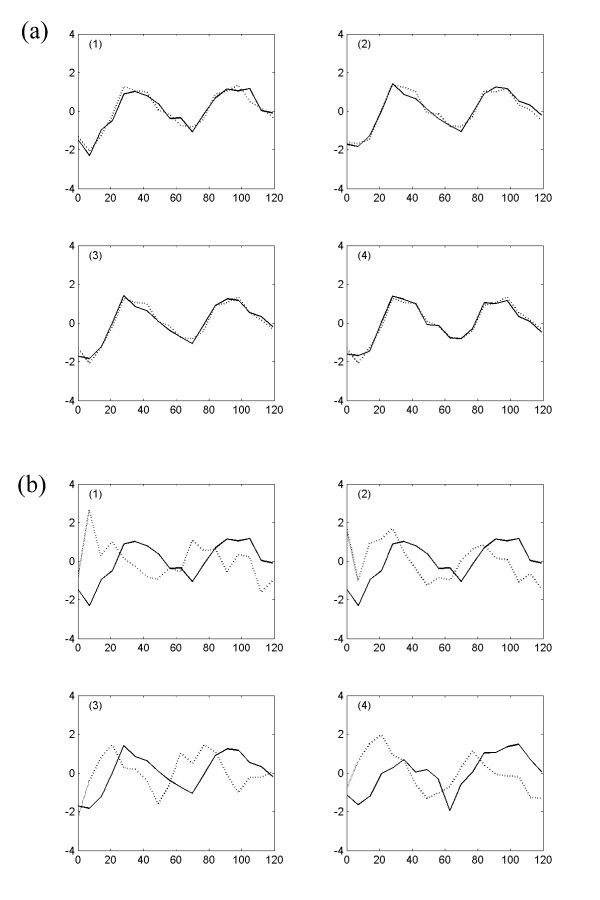
Example pairs of expression profiles from the 1^st ^cluster in the output with *cutoff *= 0.7, which are identified as known interactions from *BioGRID *database [25]. Note that these are expression profiles from the alpha-factor data set. For all figures, the x-axis corresponds to the time points for expression measurements in minutes, and y-axis expression levels. See Table 8 for systematic gene names, cell cycle phases according to the study of Spellman et al. [6], and linear correlation coefficients for each pair of genes in all figures

**Figure 10:**
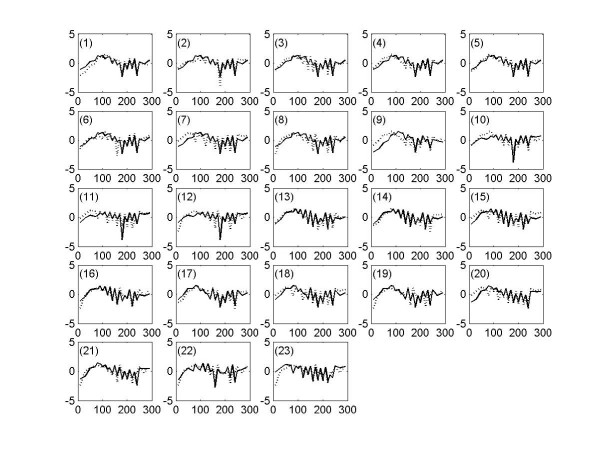
Example pairs of expression profiles from the 41^st ^cluster in the output with *cutoff *= 0.7, which are identified as known interactions from *BioGRID *database [25]. Note that these are expression profiles from the cdc15 data set. For all figures, the x-axis corresponds to the time points for expression measurements in minutes, and y-axis expression levels. See Table 9 for systematic gene names and linear correlation coefficients for each pair of genes in all figures.

To compare the capabilities of the PSC algorithm over the traditional clustering algorithm, we investigate whether genes from the output clusters are linearly correlated with each other as follows. Let's suppose that we have *n *number of genes in one of output clusters. Then, there are *(n*^2^*-n)/2 *number of all possible pairs of genes in the cluster. For each pair of genes, the linear correlation coefficients can be calculated for three expression data sets (i.e. alpha, cdc15, and cdc28), and the mean value of these three linear correlation coefficients is used as the "true" linear correlation coefficient for the given pair of genes. Note that the average values are used because of artifacts due to different cell cycle arrest treatments. Then, the mean linear correlation coefficient of all possible pairs can be obtained for each cluster, and the distribution of mean linear correlation coefficients for each output cluster with *cutoff *= 0.9, 0.8, 0.7, 0.6, 0.5 are presented in Figure 8. It is observed that the overall mean linear correlation coefficients are relatively low, and some of them are significantly low enough to be considered that their clusters are randomly created based on the linear correlation. That is, there is a significant portion of output clusters that are not obtainable by the traditional clustering algorithm. As an example, let's consider the 1^st ^cluster from the output clusters with *cutoff *= 0.7, which consists of genes associated with DNA metabolism process. From all possible pairs of genes in this cluster, we present two types of pairs: 1) similar expression profiles in Figure 9a and 2) time-shifted expression profiles in Figure 9b. It should be noted that all of these presented pairs in Figure 9 are identified as having known biological interactions between genes from the *BioGRID *database [25]. It is obvious that the time-shifted expression profiles are not obtainable by traditional clustering algorithm, because these profiles have significantly low linear correlation coefficients (<0.5). It is noteworthy that these time-shifted profiles are "similar" expression profiles that are constantly time-shifted from each other, and each gene is identified as having peak levels during different cell cycle phases according to the classification by Spellman *et al*. [6]. That is, these profiles are oscillating expression signals with a common rhythm during cell division process, which can be obtainable by PSC algorithm based on the theory of multivariate phase synchronization. As an another example, let's consider a relatively smaller cluster (41^st ^cluster from the output cluster with *cutoff *= 0.7) than 1^st ^cluster at this point. The 41^st ^cluster consisted of 11 genes, which are associated with translation process resulting in the formation of proteins. There are 23 known biological interactions identified from the *BioGRID *database [25], and these interactions are presented in Figure 10 (also see Table 9). It should be noted that none of genes in this cluster are identified as cell cycle regulated by Spellman *et al*. [6], and this example also includes expression profiles with significantly low linear correlation coefficient. This is another evidence that PSC algorithm is able to find prominent groups of genes, which are not obtainable by traditional clustering algorithm. It means that the PSC algorithm is able to find prominent groups of non-cell-cycle-regulated genes, which share significant GO terms and/or have relatively many known biological interaction from the *BioGRID *database [25]. There are more examples of such output clusters that have relative many known interactions and small (or zero) number of identified as cell-cycle-regulated genes by Spellman *et al*. [6]: e.g. 1) 3^rd^, 9^th^, 18^th^, 41^st ^clusters with *cutoff *= 0.7 (Table 4). 2) 5^th^, 19^th^, 37^th^, 50^th^, 169^th ^clusters with *cutoff *= 0.6 (Table 5) 3) 1^st^, 4^th^, 6^th^, 7^th^, 11^th^, 24^th ^clusters with cutoff = 0.5 (Table 6). It should be reminded that these clusters have relatively low linear correlation. Therefore, it suffices to say that the PSC algorithm has capabilities to find many prominent groups of genes that can not be obtained by the traditional clustering algorithms.

**Figure 11:**
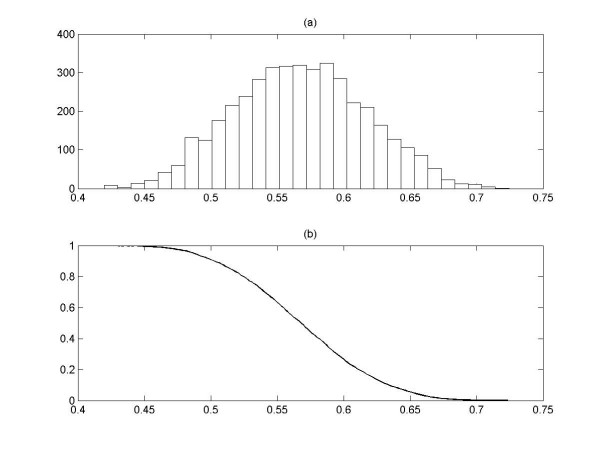
The relationship between *ρ*_*kC *_and *P-value*. (a) Top panel shows the distribution of the strength value *ρ*_*kC *_for random expression data. The x-axis corresponds to the strength value *ρ*_*kC *_and the y-axis the number of values of *ρ*_*kC *_that fall within the range of *ρ*_*kC*_. (b) The bottom panel shows how the *P-value *can be calculated by integrating the random distribution. The x-axis corresponds to the strength value *ρ*_*kC *_and the y-axis *P-value*.

For clarification of the significant of *cutoff *value, the *P-value *from the distribution of strength of phase synchronization between each oscillator (gene) and the cluster is calculated. It is reasonable to assume that the complete understanding of cellular process during cell division cycle in whole genome scale is not available yet. However, it is well known that the cell division process is a single continuous process. The cell division process is mainly consisted of *interphase *(G1, S, and G2 phase) and *division *(M phase). During the S phase, the DNA in the nucleus is replicated, and the M phase includes two separate processes, i.e. mitosis and cytokinesis. The G1 phase is an interval phase between the end of M phase and the beginning of DNA synthesis, and the G2 phase is an interval phase separating the end of DNA synthesis from the beginning of M phase. It is assumed that genes during a certain cell cycle have relatively fewer interactions with genes during the other cell cycle phase. Therefore, the cell division process is a single continuous process and each process is "weakly" connected with other process in the downstream of cell cycle process. Based on this point of view, it is assumed that the whole cell division process is consisted of genes that create a single interacting network with heterogeneous connectivity distribution; thus, whole genomes are considered to estimate the *P-value*. In order to estimate a *P-value *for a given strength of phase synchronization between each gene and the cluster *ρ*_*kC*_, a set of random expression signals is generated by shuffling the expression signals at different time points by interchanging the expression signal at time points 3 and 14. Using Eq. 9, the strength values of phase synchronization between each gene and the cluster *ρ*_*kC *_are calculated and tabulated their distribution with combined expression set of alpha-factor, cdc15 and cdc28 (Figure 11). This distribution is an approximation of true negatives for input expression signals. By integration, we could estimate a *P-value*, which is defined as the probability of obtaining a *ρ*_*kC *_larger than the *cutoff *from the random distribution: the smaller the *P-value*, the more significant the strength value *ρ*_*kC *_and vice versa.

**Figure 12:**
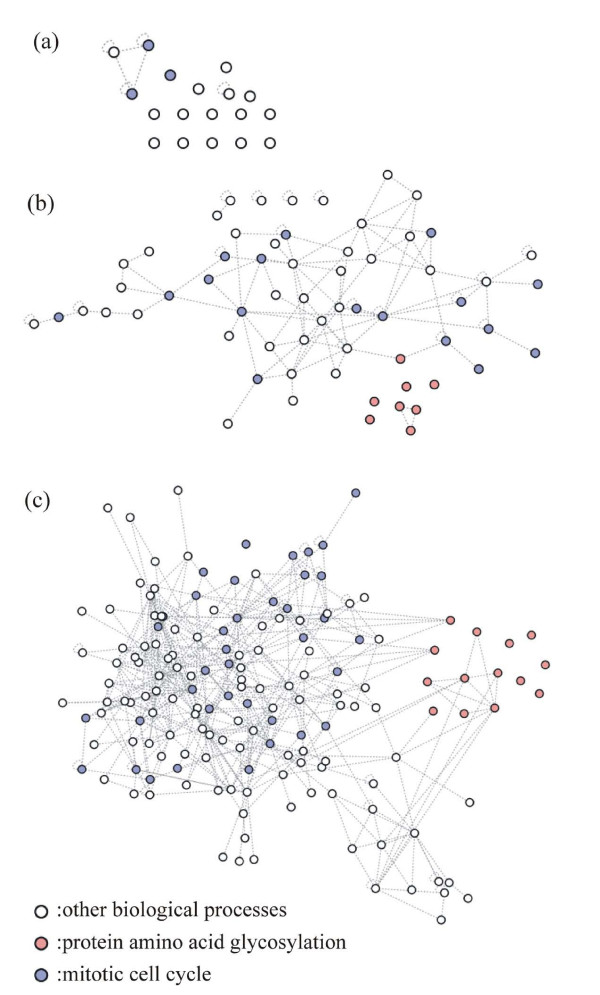
The visualization of known physical or genetic interactions from the *BioGRID *database [25] for (a) 11^th ^cluster (*cutoff *= 0.7), (b) 1^st ^cluster (*cutoff *= 0.6), and (b) 2^nd ^cluster (*cutoff *= 0.5). The nodes are labeled with color according to three GO terms of biological process: *mitotic cell cycle process*, *protein amino acid glycosylation*, and *other biological processes*.

For further understanding of the significance of *cutoff *values, we examine two biological processes (i.e. *mitotic cell cycle *and *protein amino acid glycosylation*) in three output clusters: 11^th ^cluster (*cutoff *= 0.7), 1^st ^cluster (*cutoff *= 0.6), and 2^nd ^cluster (*cutoff *= 0.5), and the known physical or genetic interactions from *BioGRID *database [25] are visualized for the selected clusters in Figure 12. It is reasonable to assume that genes associated with *mitotic cell cycle *are more "tightly" interacting with each other than the ones associated with *protein amino acid glycosylation*. Figure 12 shows that genes with *protein amino acid glycosylation *have relatively fewer known interactions than the ones with *mitotic cell cycle*. Therefore, it suffices to say that genes with *protein amino acid glycosylation *are "weakly" connected to the genes with *mitotic cell cycle*, and the genes with *protein amino acid glycosylation *are combined with genes with *mitotic cell cycle *as *cutoff *decreases. It means that the size of cluster increases as *cutoff *decreases, and the PSC algorithm creates relatively small clusters with significantly prominent genes with relatively larger *cutoff *value and these clusters grows in size by combining other "weakly" interacting genes as *cutoff *decreases. Therefore, it can be concluded that the larger the *cutoff*, the more portions of prominent genes in the output clusters and vices versa.

## Conclusion

PSC algorithm is mainly based on the theory of multivariate phase synchronization, and the phase synchronization could be understood as a common rhythm of oscillatory activities of systems due to their interactions with each other. We develop the strategy of identifying and categorizing cell cycle specific gene expressions according to the specific biological process, in which expression signals share a common rhythm during cell cycle. That is, PSC algorithm is efficient to find groups of genes that share same periodic variations of expression profiles, which is coincident with the length of the cell cycle. On the other hand, the traditional clustering algorithms search similar expression profiles with an assumption that genes with similar expression profiles have similar biological functions. Our evaluation analysis clearly indicates that PSC algorithm produces prominent clusters, which are not obtainable by traditional clustering algorithms.

Our evaluation analysis also shows that the PSC algorithm is able to find groups of gene, which are significantly associated with each other by sharing significant GO terms of biological process and/or relevant biological interactions. However, the algorithm does not have a capability to create a directed and weighted network of synchronization. Recently, Motter *et al*. [26] showed that the maximum synchronizability can be achieved when the network of synchronization is weighted and directed for a given degree distribution of heterogeneous connectivity. Therefore, the study for the analysis of cell cycle specific genome expression data could be further advanced by considering the directed and weighted network structure and addressing the effect that asymmetry has on the synchronizability of complex networks.

Based on the evaluation experiments, we draw the conclusion as follows: 1) Based on the theory of multivariate phase synchronization, it is feasible to find groups of genes, which have biological interactions and/or significantly shared GO slim terms of biological process, with cell cycle specific gene expression signals. 2) Among all the output clusters by PSC algorithm, the cluster with relatively larger size has a tendency to include more known interactions than the one with relatively smaller size. 3) It is feasible to understand the cell cycle specific gene expression patterns as the phenomenon of collective synchronization. 4) PSC algorithm is able to find prominent groups of genes, which are not obtainable by traditional clustering algorithms.

## Methods

### 1) Fundamental mathematical concept: multivariate synchronization

The proposed algorithm builds on the concepts of analytic signal and phase synchronization. Hence, we first explain the basic idea of analytic signal and phase synchronization [12, 29]. Then we continue to describe the basic idea of synchronization in ensembles of oscillating systems. By "oscillating systems", we mean systems that produce the response signals with period and frequency. As a first step, we convert the gene expression signal *x(t) *into analytic signal *x*_*a*_*(t) *using Hilbert transform (HT). The analytic signal of gene expression signal x(t) is defined by

(1) *x*_*a*_*(t) *= *x(t) *+ *jx*_*h*_(*t*)

where *j *is the imaginary unit and *x*_*h*_*(t) *is the HT of *x(t)*

(2) $$x_{h}\left(t\right)=\pi^{-1}\underset{R\to \infty }{\lim}\int_{-R}^{R}\frac{x\left(\tau \right)}{t-\tau }d\tau .$$

From this equation, it is noticed that the HT of *x(t) *may be considered as the convolution of the *x(t) *and 1/*πt*. Due to the properties of convolution, the Fourier transform (FT) *X*_*h*_*(ς) *of *x*_*h*_*(t) *is the product of the FT of *x(t) *and 1/*πt*. For physically relevant Fourier frequencies *ς *> 0, *X*_*h*_*(ς) *= -*jX(ς)*. In other words, the HT can be considered by an ideal filter whose amplitude response is unity and phase response is a constant *π*/2 lag at all Fourier frequencies. The analytic signal can also be expressed in terms of complex polar coordinates

(3) *x*_*a*_(*t*) = *A*(*t*)exp(*jφ*(*t*)),

where $A\left(t\right)=\left|x_{a}\left(t\right)\right|=\sqrt{x^{2}\left(t\right)+x_{h}^{2}\left(t\right)}$ and *φ*(*t*) = arg{*x*_*a*_(*t*)}. These two functions are respectively called the amplitude and instantaneous phase of the signal *x(t)*. The basic idea of the analytic signal is that the negative frequency components of the FT (or spectrum) of *x(t)*s are superfluous, due to the hermitian symmetry of such a spectrum. These can be removed without any loss of information, if an analytic signal is used instead. But note that the removal of the negative frequencies will eliminate such spectral symmetry; the inverse FT of such a one-sided spectrum will give back a complex analytic signal.

In this study, we use the phase of the analytic signal *x*_*a*_*(t) *to detect phase synchronization between oscillating systems; i.e. the phase synchronization can be defined as locking of the phases, while the amplitudes can be quite different. Using the methods of analytic signal, it can be shown that the interaction of nonidentical oscillators can lead to a perfect locking of their phases, whereas their amplitudes remain uncorrelated [12]. The strength of phase synchronization between two signals can be measured using the mean phase coherence [20] as follows

(4) $$\rho_{a,b}=\left|\frac{1}{n}\sum_{t}\exp\left(j\left(\phi_{a}\left(t\right)-\phi_{b}\left(t\right)\right)\right)\right|.$$

The values of *ρ*_*a*,*b *_are confined between 0 (no synchrony) and 1 (perfect synchrony) and this value monotonically increases with the strength of phase synchronization [18].

**Figure 13:**
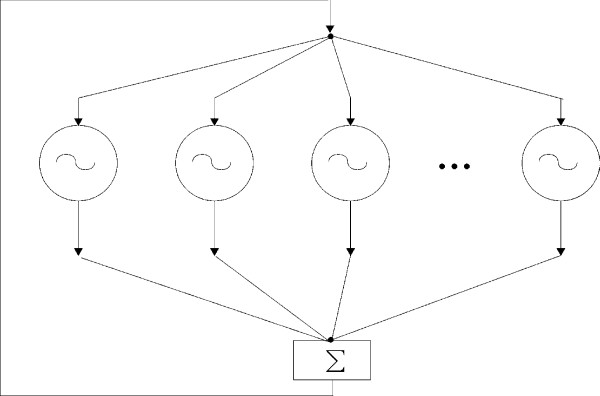
The globally coupled oscillating systems are graphically represented by Pikovsky *et al*. [11].

For multivariate oscillating systems, we use the concept of synchronization in ensembles of oscillators, in which each component interacts with all others. This can be described as *global *coupling. Let's consider an ensemble of non-identical oscillators to understand the process of collective synchronization. From the previous section, it is understood that a pair of systems can be synchronized, and it is expected that synchronization can be extended to a whole population of systems, or at least to a large portion of it. Pikovsky *et al*. [11] explained how such coupling results in synchronization in the ensemble with a drawing as shown in Figure 13. With this figure, they have described the driving inputs that come to each system from all others by one input from the whole ensemble. It means that a common force operates each system and this force is proportional to the sum of outputs of all systems in the ensemble. This force can entrain many oscillating systems if their frequencies are close. In the case of global coupling this force is not predetermined, but comes from interaction within the ensemble. To explain qualitatively this force, we consider the study of Allefeld and Kurths [17]. They described the basic idea of multivariate synchronization analysis to understand the oscillating systems as constituting a cluster, in which each component system participates in different degree. The cluster consists of a common rhythm and it is described by the dynamics of a cluster phase Φ. For each measurement, the phase of the cluster is defined as a circular weighted mean of all phases inside the cluster,

(5) $$\Phi \left(t\right)=\arg\sum_{m}c_{m}\exp\left(j\phi_{m}\left(t\right)\right),$$

where the participation index *c*_*m *_can be obtained as a function of the synchronization strength between a system and the cluster,

(6) $$\begin{array}{ccc} {\text{c}}_{\text{m}}=f\left(\rho_{\text{m},\text{C}}\right) & \text{with} & \rho_{\text{m},\text{C}}\left|\frac{1}{n}\sum_{t}\exp\left(j\left(\phi_{m}\left(t\right)-\Phi \left(t\right)\right)\right)\right| \end{array}.$$

This participation index *c*_*m *_measure both how close each system inside the cluster follow the common rhythm Φ as well as how much a system contribute to the cluster. In this definition, it is not clear which function *f *should be chosen for the relationship between the *ρ*_*m*,*C *_and the *c*_*m*_. They modified the idea of synchronization cluster analysis in a way that is much more generally applicable. For the strength values of the bivariate synchronization *ρ*_*a*,*b *_can be defined as

(7) $$\begin{array}{c} \rho_{a,b}=\left|\langle \exp\left(j\left(\phi_{a}-\phi_{b}\right)\right)\rangle \right|\\ =\left|\langle \exp\left(j\left(\Delta \phi_{a}-\Delta \phi_{b}\right)\right)\rangle \right|, \end{array}$$

where Δ*φ*_*a *_= *φ*_*a *_- Φ and Δ*φ*_*b *_= *φ*_*b *_- Φ. They introduce the phase of the mean field to factorize the strength of phase synchronization as follow,

(8) $$\begin{array}{c} \rho_{a,b}=\left|\langle \exp\left(j\Delta \phi_{a}\right)\rangle \right|\left|\langle \exp\left(-j\Delta \phi_{b}\right)\rangle \right|\\ =\rho_{a,C}\rho_{b,C}, \end{array}$$

where *a *≠ *b *(*ρ*_*aa *_= 1). This factorization makes possible to estimate the strength of the synchronization between a system and the cluster, *ρ*_*aC*_, thus this leads to the derivation of an iteration equation for estimating *ρ*_*kC *_as follow

(9) $${\rho^{\prime }}_{kC}=\frac{1}{2}\left(\rho_{kC}+\frac{\sum_{i}F_{ik}\rho_{iC}\rho_{ik}}{\sum_{i}F_{ik}\rho_{iC}^{2}}\right),$$

with *F*_*ik *_= 1/(1 - *ρ*_*iC*_^2^*ρ*_*ik*_^2^)^2 ^for *i *≠ *k *and *F*_*ik *_= 0 for *i *= *k*.

### 2) Phase synchronization clustering algorithm

Based on the concept of synchronization in ensembles of oscillating systems, we propose the strategy to make clusters of genes based on the theory of multivariate synchronization. There are 5 steps in this procedure. The descriptions for each step are listed as follow. Inputs to this method are the time series of expression data set and *cutoff *value for synchronization strength.

#### Step 1. Obtaining the phase vector *φ*_*i*_

Let's say there are signals *x*_*i*_*(t) *of the *K *systems *i *= 1, 2, ..., *K *with n number of observations *t *= 1, 2, ..., *n *of the stochastic process. In this step, the analytical signal can be approximated using Fast Fourier transform [27]. The output of this step is phase vector *φ*_*i*_, which is defined as *φ*_*i *_= {*φ*_*i*_*(1)*, *φ*_*i*_*(2)*, ..., *φ*_*i*_*(n)*}, for 1 ≤ *i *≤ *K*.

#### Step 2. Initialization of cluster array

First, an array for *K *number of clusters is produced. For each cluster *cluster(i)*, a phase vector *φ*_*i *_is stored for 1 ≤ *i *≤ *K*. The output of this step is cluster array *cluster(i)*, for 1 ≤ *i *≤ *K*. The pseudo algorithm of this step is presented in APPENDIX A.

#### Step 3. Initial clustering

For each phase vector, this step finds how closely the phase vector follows the common rhythm for each cluster from the array. This can be measured by the synchronization strength between the phase vector and the cluster. Then the algorithm finds the cluster in which the phase vector has the highest value of the synchronization strength. If the synchronization strength between the phase vector and the selected cluster is greater or equals to the pre-defined *cutoff *value, this cluster is updated by appending the phase vector to the selected cluster. This procedure is repeated for the entire phase vectors. The output of this step is the updated cluster array. The pseudo algorithm of this step is presented in APPENDIX B.

#### Step 4. Filtering cluster

If the cluster contains no more than a system, this does not constitute as a cluster. Thus, the cluster is set to empty list. The pseudo algorithm of this step is presented in APPENDIX C.

#### Step 5. Combining clusters

Empty clusters are not considered in this step. For each non-empty cluster, the algorithm finds a cluster from the array such that these two clusters will have the most common rhythm when they are combined. If all of the synchronization strength between the combined cluster and each element are greater or equals to the *cutoff *value, these two clusters are combined. The pseudo algorithm is presented in APPENDIX D.

## Appendix A: The pseudo algorithm for *initialization of cluster array*

Input: phase vectors, *φ*_*i *_for 1 ≤ i ≤ K.

Output: cluster array, *cluster(i) *for 1 ≤ i ≤ K.

for 1 ≤ i ≤ K

   *cluster(i) *= {*φ*_i_}

end

## Appendix B: The pseudo algorithm for *initial clustering*

Input: *cutoff *and cluster array, *cluster(i) *for 1 ≤ i ≤ K.

Output: cluster array, *cluster(i) *for 1 ≤ i ≤ K.

for 1 ≤ i ≤ K

   Initialize *[SynStrength]*^*(j) *^with 0, for 1 ≤ j ≤ K

   for 1 ≤ j ≤ K, i ≠ j

      *temp_list *= {*cluster(j)*, *φ*_*i*_}

      n = the size of *temp_list*

      Compute *ρ*_mC _using *φ*_*m *_from *temp_list*, for 1 ≤ m ≤ n

      *[SynStrength]*^*(j) *^= *ρ*_nC_

   end

   Find *max_SynStrength *= max{*[SynStrength]*^*(j)*^, 1 ≤ j ≤ K, i ≠ j}

   If *max_SynStrength *≥ *cutoff*

      *cluster(j) *= {*cluster(j)*, *φ*_*i*_}

   end

end

## Appendix C: The pseudo algorithm for *filtering cluster*

Input: cluster array, *cluster(i) *for 1 ≤ i ≤ K.

Output: cluster array, *cluster(i) *for 1 ≤ i ≤ K.

for 1 ≤ i ≤ K

   if the size of *cluster(i) *equals to 1

      *cluster(i) *= {}

   end

end

## Appendix D: The pseudo algorithm for *combining clusters*

Input: *cutoff *and cluster arrays, *cluster(i) *for 1 ≤ i ≤ K.

Output: cluster array, *cluster(i) *for 1 ≤ i ≤ K.

for 1 ≤ i ≤ K

   Initialize *[SynStrength]*^*(j) *^with 0, for 1 ≤ j ≤ K

   for 1 ≤ j ≤ K, i ≠ j

      if *cluster(i) *and *cluster(j) *are not empty lists

         *temp_list *= {*cluster(j)*, *cluster(i)*}

         n = the size of *temp_list*

         Compute *ρ*_mC _using *φ*_*m *_from *temp_list*, for 1 ≤ m ≤ n

         *[SynStrength]*^*(j) *^= min{*ρ*_mC_, 1 ≤ m ≤ n}

      end

   end

   Find *max_SynStrength *= max{*[SynStrength]*^*(j)*^, 1 ≤ j ≤ K, i ≠ j}

   If *max_SynStrength *≥ *cutoff*

      *cluster(j) *= {*cluster(j)*, *cluster(i)*}

      *cluster(i) *= {}

   end

end

## Authors' contributions

CSK conceived this study, developed the proposed method, and performed the evaluation experiments. CSK, CSB, and HJT participated in the interpretation of the evaluation experiments. CSK, CSB and HJT drafted and finalized the manuscript. All authors approved the manuscript.

**Table 7:** The index for each figure depending on *cutoff *and *noise level ε *in Figure 3.

Figures	*noise level ε*	*cutoff*
(a)	0.1	0.6
(b)	0.1	0.7
(c)	0.1	0.8
(d)	0.1	0.9
(e)	0.2	0.6
(f)	0.2	0.7
(g)	0.2	0.8
(h)	0.2	0.9
(i)	0.3	0.6
(j)	0.3	0.7
(k)	0.3	0.8
(l)	0.3	0.9
(m)	0.4	0.6
(n)	0.4	0.7
(o)	0.4	0.8
(p)	0.4	0.9

**Table 8:** Systematic gene names, cell cycle phases according to the study of Spellman et al. [6], and linear correlation coefficients for each pair of genes in Figure 9.

	Solid lines		Dotted lines		
		
Index	Gene name	Cell cycle	Gene name	Cell cycle	linear correlation coefficient
(a)					
(1)	YBL003C	S phase	YDR225W	S phase	0.9102
(2)	YBR010W	S phase	YDR224C	S phase	0.9441
(3)	YBR010W	S phase	YDR225W	S phase	0.9422
(4)	YDR224C	S phase	YDR225W	S phase	0.9619
(b)					
(1)	YBL003C	S phase	YIL026C	G1 phase	0.3129
(2)	YBL003C	S phase	YNL262W	G1 phase	0.2275
(3)	YBR010W	S phase	YML102W	G1 phase	0.1558
(4)	YDL055C	G1 phase	YER095W	G1 phase	0.4358

**Table 9:** Systematic gene names, and linear correlation coefficients for each pair of genes in Figure 10.

Index	solid line	dotted line	linear correlation coefficient	index	solid line	dotted line	linear correlation coefficient
1	YGL103W	YGL123W	0.64664	13	YHR203C	YJL177W	0.65787
2	YGL103W	YGL147C	0.60397	14	YHR203C	YLR075W	0.60341
3	YGL103W	YHR203C	0.53591	15	YHR203C	YML063W	0.67265
4	YGL103W	YJL177W	0.5584	16	YHR203C	YOL127W	0.50694
5	YGL103W	YLR075W	0.51294	17	YJL177W	YLR075W	0.75556
6	YGL103W	YLR325C	0.45147	18	YJL177W	YML063W	0.75692
7	YGL103W	YML063W	0.43962	19	YJL177W	YOL127W	0.47048
8	YGL103W	YOL127W	0.6723	20	YLR075W	YML063W	0.76566
9	YGL123W	YJL177W	0.70109	21	YLR075W	YOL127W	0.47712
10	YGL147C	YJL177W	0.70483	22	YLR325C	YOL127W	0.47861
11	YGL147C	YML063W	0.72561	23	YML063W	YOL127W	0.46216
12	YGL147C	YOL127W	0.40261				

## Supplementary Material

Additional file 1 Tables of significant GO terms of biological process for the output clusters with cutoff = 0.9. This file includes 8 clusters from the PSC algorithm with cutoff = 0.9, which have significantly GO terms of biological process. These significant GO terms are obtained by a tool called *GO Term Finder*, which is available from *Saccharomyces *Genome Database [24]. Click here for file

Additional file 2 Tables of significant GO terms of biological process for the output clusters with cutoff = 0.8. This file includes 57 clusters from the PSC algorithm with cutoff = 0.8, which have significantly GO terms of biological process. These significant GO terms are obtained by a tool called *GO Term Finder*, which is available from *Saccharomyces *Genome Database [24]. Click here for file

Additional file 3 Tables of significant GO terms of biological process for the output clusters with cutoff = 0.7. This file includes 148 clusters from the PSC algorithm with cutoff = 0.7, which have significantly GO terms of biological process. These significant GO terms are obtained by a tool called *GO Term Finder*, which is available from *Saccharomyces *Genome Database [24]. Click here for file

Additional file 4 Tables of significant GO terms of biological process for the output clusters with cutoff = 0.6. This file includes 151 clusters from the PSC algorithm with cutoff = 0.8, which have significantly GO terms of biological process. These significant GO terms are obtained by a tool called *GO Term Finder*, which is available from *Saccharomyces *Genome Database [24]. Click here for file

Additional file 5 Tables of significant GO terms of biological process for the output clusters with cutoff = 0.5. This file includes 21 clusters from the PSC algorithm with cutoff = 0.8, which have significantly GO terms of biological process. These significant GO terms are obtained by a tool called *GO Term Finder*, which is available from *Saccharomyces *Genome Database [24]. Click here for file
